# Is Limb Salvage With Microwave-induced Hyperthermia Better Than Amputation for Osteosarcoma of the Distal Tibia?

**DOI:** 10.1007/s11999-017-5273-1

**Published:** 2017-02-13

**Authors:** Kang Han, Peiye Dang, Na Bian, Xiang Chen, Tongtao Yang, QingYu Fan, Yong Zhou, Tingbao Zhao, Pingshan Wang

**Affiliations:** 1 Department of Spinal Cord Injury, General Hospital of Jinan Military Area Command of Chinese PLA, Jinan, 250000 Shandong People’s Republic of China; 2 Department of Orthopedic Surgery Center, Xingyuan Hospital, Yulin, Shaanxi People’s Republic of China; 3 Department of Pediatrics, Baylor College of Medicine, Houston, TX 77030 USA; 4 Department of Orthopedic Surgery, Orthopedics Oncology Institute of Chinese PLA, Tangdu Hospital, Fourth Military Medical University, Xi’an, Shaanxi People’s Republic of China

## Abstract

**Background:**

Amputation has been the standard surgical treatment for distal tibia osteosarcoma owing to its unique anatomic features. Preliminary research suggested that microwave-induced hyperthermia may have a role in treating osteosarcoma in some locations of the body (such as the pelvis), but to our knowledge, no comparative study has evaluated its efficacy in a difficult-to-treat location like the distal tibia.

**Questions:**

Does microwave-induced hyperthermia result in (1) improved survival, (2) decreased local recurrence, (3) improved Musculoskeletal Tumor Society (MSTS) scores, or (4) fewer complications than amputation in patients with a distal tibial osteosarcoma?

**Methods:**

Between 2000 and 2015, we treated 79 patients for a distal tibia osteosarcoma without metastases. Of those, 52 were treated with microwave-induced hyperthermia, and 27 with amputation. Patients were considered eligible for microwave-induced hyperthermia if they had an at least 20-mm available distance from the tumor edge to the articular surface, good clinical and imaging response to neoadjuvant chemotherapy, and no pathologic fracture. Patients not meeting these indications were treated with amputation. In addition, if neither the posterior tibial artery nor the dorsalis pedis artery was salvageable, the patients were treated with amputation and were not included in any group in this study. A total of 13 other patients were treated with conventional limb-salvage resections and reconstructions (at the request of the patient, based on patient preference) and were not included in this study. All 79 patients in this retrospective study were available for followup at a minimum of 12 months (mean followup in the hyperthermia group, 79 months, range 12–158 months; mean followup in the amputation group, 95 months, range, 15–142 months). With the numbers available, the groups were no different in terms of sex, age, tumor grade, tumor stage, or tumor size. All statistical tests were two-sided, and a probability less than 0.05 was considered statistically significant. Survival to death was evaluated using Kaplan-Meier analysis. Complications were recorded from the patients’ files and graded using the classification of surgical complications described by Dindo et al.

**Results:**

In the limb-salvage group, Kaplan Meier survival at 6 years was 80% (95% CI, 63%–90%), and this was not different with the numbers available from survivorship in the amputation group at 6 years (70%; 95% CI, 37%–90%; p = 0.301).With the numbers available, we found no difference in local recurrence (six versus 0; p = 0.066). However mean ± SD MSTS functional scores were higher in patients who had microwave-induced hyperthermia compared with those who had amputations (85% ± 6% versus 66% ± 5%; p = 0.008).With the numbers available, we found no difference in the proportion of patients experiencing complications between the two groups (six of 52 [12%] versus three of 27 [11%]; p = 0.954).

**Conclusions:**

We were encouraged to find no early differences in survival, local recurrence, or serious complications between microwave-induced hyperthermia and amputation, and a functional advantage in favor of microwave-induced hyperthermia. However, these findings should be replicated in larger studies with longer mean duration of followup, and in studies that compare microwave-induced hyperthermia with conventional limb-sparing approaches.

**Level of Evidence:**

Level III, therapeutic study.

## Introduction

The tibia is the second most-common site of osteosarcoma, accounting for 19% of all osteosarcomas, with 20% of those occurring in the distal tibia [22]. Amputation has long been regarded as the standard surgical treatment for these tumors, with satisfactory functional results when an appropriate prosthesis is used [25]. With the advances in chemotherapy and surgical techniques, limb salvage has become the preferred treatment when possible. However, other than in locations like those surrounding the hip or knee, it is difficult to perform a safe, negative-margin resection in the distal tibia because of its subcutaneous location and the proximity of the distal tibia to the neurovascular bundle and tendons [18]. Complications, poor function, and decreased durability of the reconstruction are difficult to avoid in this location [19].

Conflicting findings regarding survival and function after limb salvage and amputation for patients with osteosarcoma of the distal tibia have been reported [2, 4, 15, 19, 20, 26]. While survivorship of patients who undergo amputation for distal tibia osteosarcoma generally is high [26] and complications are disconcertingly frequent [4], function as measured by the Musculoskeletal Tumor Society (MSTS) [6] score after amputation is generally low [15]. Small series of patients undergoing limb salvage for osteosarcoma in this location are not always dramatically better in terms of function [20], complications are likewise common [25], and survivorship seems even worse [18]. For this reason, we believe the best surgical option for patients who have osteosarcoma of the distal tibia is unclear.

Hyperthermia has been introduced as an alternative treatment method for osteosarcoma [8]. It is capable of accurately killing tumor cells while tending to minimize injury to the surrounding tissue, perhaps facilitating resections in difficult-to-access locations. Hyperthermia can be used to achieve acceptable local disease control while maintaining the structural integrity of the skeleton in some patients [9]. This technique may reduce the need for complex reconstruction, and so seems appealing in terms of potential functional benefits; however, this is unproven for patients with osteosarcoma of the distal tibia. In this setting, microwave-induced hyperthermia is administered to the tumor bed and causes immediate heat necrosis of the tumor and adjacent tissues, followed by limited surgical excision of the mass with preservation of the surrounding skeleton. Because of its perceived benefits, we have used microwave-induced hyperthermia in patients with malignant bone tumors for 20 years in our department [7, 8]; however, no formal study has compared microwave-induced hyperthermia with the conventional treatment (transtibial amputation), and it seems important to do so.

We therefore asked: Does microwave-induced hyperthermia result in (1) improved survival, (2) decreased local recurrence, (3) improved MSTS scores, or (4) fewer complications than amputation in patients with a distal tibial osteosarcoma?

## Patients and Methods

The research was approved by the Ethics Review Committee of Tangdu Hospital, Xian, Shanxi, China (approval ID 2016016), and written informed consent was obtained from all participating patients.

### Cohort Selection

Between 2000 and 2015, we treated 106 patients for distal tibia osteosarcoma without metastases. Of those, 52 were treated with microwave-induced hyperthermia (Table 1), and 41 with amputation. A total of 13 patients who would have met our indications for microwave-induced hyperthermia were instead treated with the conventional limb-salvage resection and reconstruction based on the patient’s preferences (which might have been driven by cost, perceived functional demand, or other factors); these patients were not included in this retrospective study. If neither the posterior tibial artery nor the dorsalis pedis artery was salvageable, patients were treated with amputation; these patients (n = 14) were not included in any group in this study. This left 27 patients with amputations available for our study (Table 2) and 79 patients available for the entire study. Patients were considered eligible for microwave-induced hyperthermia if they had an at least 20 mm available distance from the tumor edge to the articular surface, good clinical (such as pain reduction) and imaging responses to neoadjuvant chemotherapy, and no pathologic fracture. Chemonecrosis was assessed using the grading system of Huvos et al. [14]. More than 90% necrosis on the histologic sections was considered a good response to chemotherapy. All patients in this series were available for followup at a minimum of 12 months (mean followup in the hyperthermia group, 79 months, range, 12–158 months; mean followup in the amputation group, 95 months, range, 15–142 months).

**Table 1:** Details of the patients with distal tibia osteosarcoma who had microwave-induced hyperthermia

Patient number/ gender/age (years)	AJCC stage	Histology/Broders’ grade	Tumor size (cm^2^)	Chemotherapy
1/F/31	IIA	3	11.6	+
2/M/9	IIA	4	8.8	+
3/M/28	IIB	4	9.5	+
4/F/20	IB	3	12.3	+
5/F/33	IIA	4	4.6	+
6/M/25	IIB	2	13.6	+
7/F/53	IB	4	12.8	+
8/M/26	IIA	4	11.6	+
9/M/50	IB	1	9.1	+
10/F/12	IIB	4	20.6	
11/M/21	IIA	2	12.5	+
12/F/23	IB	4	15.2	+
13/M/32	IIA	2	11.4	+
14/F/25	IIB	4	12.5	+
15/F/23	IB	1	7.8	+
16/M/34	IIA	4	6.6	+
17/M/23	IIB	3	13.2	+
18/F/44	IB	4	14.6	+
19/M/39	IIB	1	15.1	+
20/F/40	IIB	4	12.3	+
21/M/38	IA	4	8.9	+
22/M/18	IIA	2	9.7	+
23/F/14	IIA	4	6.8	+
24/M/20	IIB	3	15.2	+
25/M/30	IIA	2	9.4	+
26/M/52	IA	4	8.3	+
27/F/8	IIA	4	8.1	+
28/M/32	IIA	2	7.6	+
29/F/23	IA	4	15.5	+
30/M/27	IIA	3	13.1	+
31/F/20	IIB	1	16.8	+
32/M/34	IIA	4	8.7	+
33/F/25	IIA	3	13.3	+
34/M/20	IA	2	4.2	+
35/M/44	IIB	4	8.8	+
36/F/27	IIA	4	12.7	+
37/M/17	IIA	1	21.2	+
38/F/18	IIA	4	12.6	+
39/M/25	IA	3	5.3	+
40/F/33	IIA	4	15.6	+
41/M/30	IIA	4	13.9	+
42/F/24	IIA	3	15.4	+
43/M/30	IA	4	13.2	+
44/M/45	IIA	4	12.5	+
45/F/28	IIA	3	15.2	+
46/M/28	IIA	4	14.7	+
47/F/12	IA	4	17.6	+
48/M/21	IIA	3	18.6	+
49/M/34	IIA	4	12.9	+
50/M/27	IIA	3	15.4	+
51/F/27	IIA	3	17.8	+
52/M/40	IA	3	16.2	+

AJCC = American Joint Committee on Cancer.

**Table 2:** Details of the patients with distal tibia osteosarcoma who had amputation

Patient number/ gender/age (years)	AJCC stage	Histology/Broders’ grade	Tumor size (cm^2^)	Chemotherapy
1/M/21	IIB	4	10.4	+
2/M/42	IB	2	8.4	−
3/M/34	IIB	4	12.9	+
4/F/26	IIB	3	11.1	+
5/M/28	IIA	3	9.2	−
6/F/32	IIB	3	8.5	−
7/F/15	IIA	4	11.5	+
8/F/24	IIB	4	10.6	+
9/M/16	IIB	4	9.7	−
10/F/35	IIB	3	18.2	
11/F/34	IIA	4	21.3	+
12/M/25	IIB	4	13.2	+
13/M/30	IIA	4	19.4	+
14/F/33	IIA	4	10.9	+
15/M/27	IIB	4	19.3	+
16/F/52	IIA	4	12.7	+
17/M/33	IB	3	11.2	+
18/F/30	IIB	4	14.6	+
19/F/29	IB	1	5.4	+
20/M/13	IIB	4	17.2	+
21/M/34	IIA	4	9.9	+
22/F/21	IIB	4	9.6	−
23/F/40	IB	3	12.5	+
24/F/25	IIB	3	17.6	+
25/F/27	IIA	4	9.1	+
26/M/27	IB	3	8.6	−
27/F/30	IIB	4	8.4	−

AJCC = American Joint Committee on Cancer.

All patients had radiographs, CT, MRI, and bone scans. With the numbers available, we found no difference in sex between the amputation group and microwave-induced hyperthermia group (12 males and 15 females versus 30 males and 22 females; p = 0.263) (Table 3). We also found no difference in age (27.5 ± 8.7 years versus 31.2 ± 6.4 years; p = 0.586), tumor grade, tumor stage, and tumor size between the amputation group and the microwave-induced hyperthermia group (Table 3). Of the 79 patients, 54 had a needle biopsy and 32 had an incisional biopsy, including those whose needle biopsy was nondiagnostic. We graded the histologic sections based on the biopsy using Broders’ classification [1], which has four grades according to the rate of differentiation of the tumor cells. We staged patients using the surgical staging systems of the MSTS [6] and the American Joint Committee on Cancer (AJCC) [24]. Nineteen patients had Stage I tumors and 60 had Stage II tumors.

**Table 3:** Comparison of the clinical information, clinical efficacy, and incidence of complications between two groups

Variable	Limb salvage (n = 52)	Amputation (n = 27)	p Value	Statistical test
Sex (male:female)	30:22	12:15	0.263	Chi-square
Age (years)	27.5 ± 8.7	31.2 ± 6.4	0.586	Student’s t-test
Tumor grade			0.083	Chi-square
Grades 1 and 2	12	2		
Grades 3 and 4	40	25		
Tumor stage			0.407	Chi-square
Stage IA or IB	14	5		
Stage IIA or IIB	38	22		
Tumor size				
≤ 8 cm^2^	7	1	0.095	Mann-Whitney U test
> 8 cm^2^	45	26		
Followup	Mean, 75.3 months	Mean, 51.2 months		
Local recurrence	6	0	0.066	Chi-square
MSTS functional score	85.3 ± 5.5	65.9 ± 4.9	0.008*	Student’s t-test
Complications	6	3	0.954	Chi-square

*Statistically significant.

### Surgical Technique

All patients were evaluated by CT and MRI at the end of each chemotherapy regimen preoperatively to define the edge of the tumor, which was determined at the transition of marrow signal from abnormal to normal. Areas of intermediate signal intensity adjacent to the tumor edge were regarded as part of the tumor and should be included in the ablation area. All 79 patients received two cycles of preoperative neoadjuvant chemotherapy based on a standard protocol which was described in a previous study [17].

Patients treated with microwave-induced hyperthermia were evaluated according to the following criteria: (1) assessment of tumor response or progression as assessed by MRI; (2) distance between the ankle cartilage and the tumor as assessed by MRI of 20 mm or more, to obtain a bone width margin of 10 mm and a remaining residual epiphysis of 10 mm, and wide proximal margins on the bone resections [19] (defined as a cuff of 2 cm to 3 cm of normal tissue remaining on all sides of the tumor); and (3) a sufficient amount of epiphysis preserved to allow fixation of the osteotomy junction [21]. Intraoperatively, the adequacy of bone resection was evaluated with frozen section biopsy of a tissue sample obtained from the medullary canal of the residual tibia. For all patients who had amputations, the margins were wide (a cuff of 2 cm to 3 cm of normal tissue remaining on all sides of the tumor). After surgery, the histologic margins were negative in all patients.

**Fig. 1A–L:**
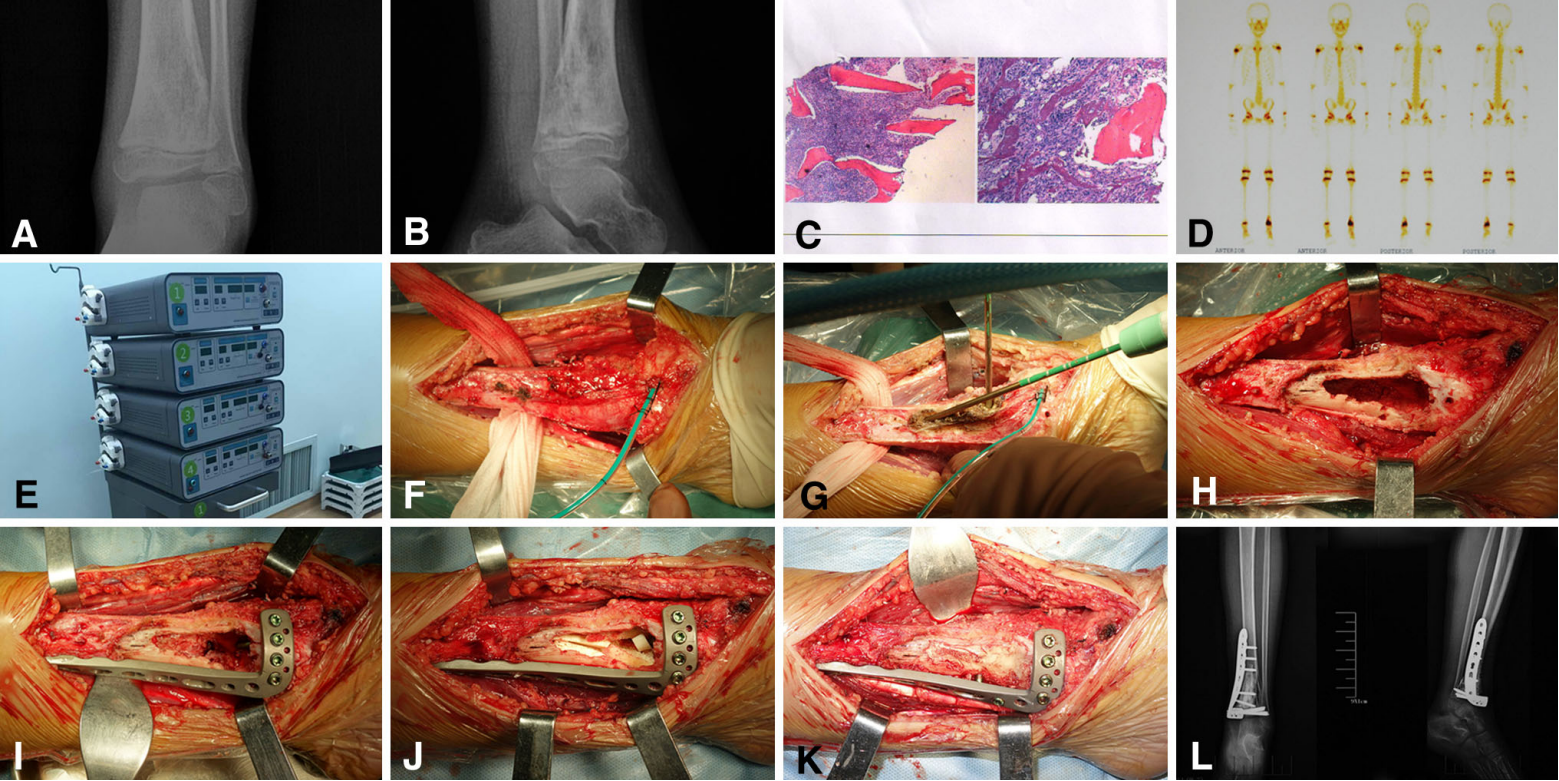
(**A**) AP and (**B**) lateral radiographs show an osteosarcoma of the distal tibia. (**C**) Pathologic examination of the tumor, and (**D**) bone scans are shown. (**E**) The photograph shows the microwave-induced hyperthermia machine. (**F**) Dissection is shown of the tumor-bearing bone from the surrounding normal tissues with a safe margin. (**G**) A wet gauze is placed between the tumor bone and surrounding normal tissues and electromagnetic energy is delivered to the tumor bone. (**H**) The dead or softened tumor is removed or curetted, (**I**) followed by prophylactic fixation. (**J**) The defect then is filled with allograft bone, (**K**) as shown in this image of the mixture of bone cement and allograft bone chips. (**L**) Postoperative AP and lateral radiographs are shown.

All operations were performed by the same two surgeons (QYF and YZ). The microwave-induced hyperthermia machine we used was the FORSEA (Xinhua Company, Nanjing, China) [9, 10], and the microwave generator frequency is 2450 MHz. When microwave-induced hyperthermia was performed (Fig. 1), the main principle was to dissect the tumor with a safe margin as described above, and subsequently perform an en bloc ablation using antenna-guided hyperthermia therapy. The first step was to identify the extent of the tumor and dissect the tumor-bearing bone from surrounding normal tissues with a safe margin (at least 20 mm width). We usually use the original dissection method of double incisions to obtain adequate exposure (Fig. 1F). This step is very important because it is helps to ensure the entire tumor can be killed by microwave-induced hyperthermia. A heat-isolation pad and wet gauze then were placed between the bone tumor and surrounding normal tissues. Then, one to six antennas were placed in different location of the tumor from different angles, matching the suction one-to-one, according to the shape and size of the tumor to ensure the therapeutic range and the tumor edge could be ablated adequately. Heat output was instant when the antennas were placed, and electromagnetic energy then was delivered to the tumor (Fig. 1G). The tumor was ablated with direct heating while normal soft tissues were protected from overheating. The goal of microwave ablation is to create an ablation zone that extends 1 cm beyond the tumor boundary at all points with the core temperature of the tumor reaching 85° to 100°C and the normal tissue temperature remaining less than 40°C for 15 to 20 minutes. During surgery, a circulating cool saline system was used to protect the surrounding normal tissues, and multiple thermocouples were placed in various critical locations to monitor the temperature. To avoid damaging the joint, outlet piping which was connected with a circulating water pump and the thermocouples were specifically placed in the ankle cavity to keep the articular cartilage and its subchondral bone from overheating. All the tissue blocks were evaluated histologically for tumor hyperthermia necrosis and the histologic examination showed that part of the proximal margins were histologically negative and part of the margins were necrotic. After the dead tumor mass was removed or curetted (Fig. 1H), the reconstruction was performed using a mixture of bone chips and bone cement (Fig. 1I–K) [18]. The normal shape of the tibia was restored and prophylactic fixation was performed if necessary (Fig. 1I–K).

Transtibial amputation was performed as common practice [13].The goals and requirements were resecting the bone 2 cm to 3 cm proximal to abnormal bone density, obtaining adequate length of the residual limb, and achieving good soft tissue coverage.

### Postsurgery Rehabilitation and Followup

All patients in both groups were given antibiotics for 72 hours after surgery, and they performed bed exercises until wound healing was achieved. A short cast or a brace was used for patients who had microwave-induced hyperthermia until there was radiographic evidence of bone union. Signs of bony union were evaluated by serial sets of plain radiographs [11]. All patients in both groups received postoperative chemotherapy (adriamycin, cisplatin, methotrexate, ifosfamide) [10]).

After discharge from the hospital, clinical and radiographic followups are done every month during the first 6 months, then every 3 months during the next 2 years, and then every 6 months. Chest CT scans were performed to observe pulmonary metastasis every 3 months during the first year and then every 6 months afterward. A bone scan was performed every 6 months during the first year and then every year. All patients have radiographs taken once a year. The MSTS score was used to observe the function of the patients. The status and function of the ankle were specifically assessed clinically and radiologically at followups.

### Clinical Outcomes

Clinical outcomes were assessed by review of clinic notes, supplemented by phone questionnaires, and email where needed. Local recurrence, metastasis, complications, and death were recorded from the patients’ files. Complications were graded using the classification described by Dindo et al. [3], which graded the complications at five levels. Followup review and data were sorted and analyzed by three of the authors (KH, NB, TY).

### Statistical Analysis

All values are expressed as mean ± SD, and all error bars represent the SD of the mean. Student’s t test and one-way ANOVA were used to determine significance. Survival rates were estimated using the Kaplan-Meier method. We compared survival between the two groups using a log-rank test. Chi-square test was used to compare complications between the two groups. The mean, SD, and 95% CI were provided. All statistical tests were two-sided. A probability less than 0.05 was considered statistically significant. Statistical analyses were performed using SPSS Version 17.0 (SPSS Inc, Chicago, IL, USA).

## Results

### Survival

**Fig. 2:**
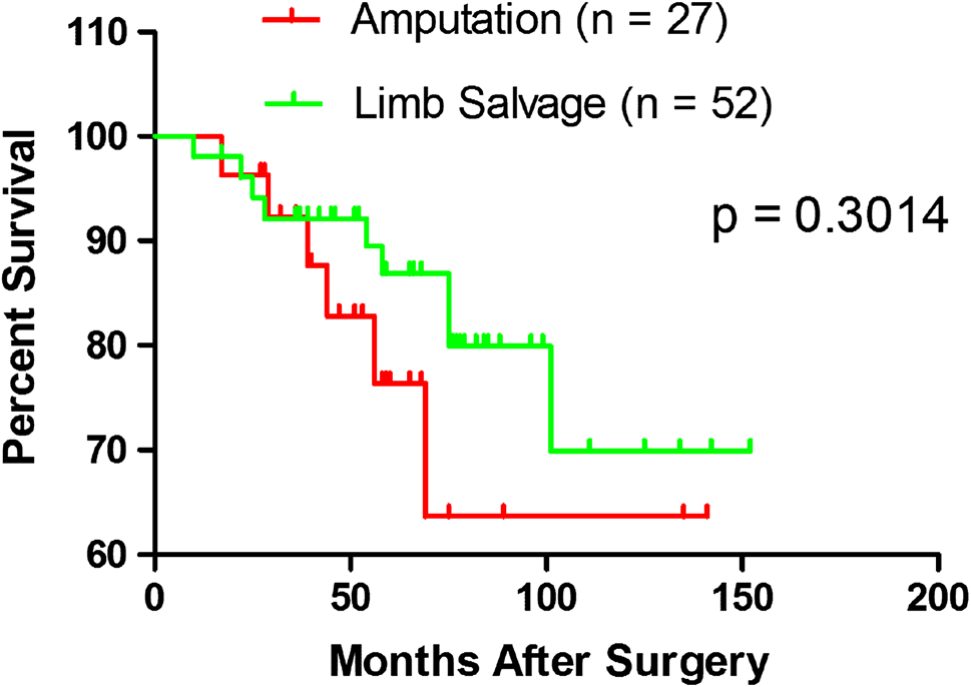
A graph shows similar (log rank tests, p = 0.3014) survival for patients with distal tibia osteosarcomas treated with microwave-induced hyperthermia and with amputation.

With the numbers available, there was no difference in Kaplan-Meier survivorship between the groups. In the limb salvage group, Kaplan Meier survival at 6 years was 80% (95% CI, 63%–90%), and in the amputation group it was 70% at 6 years (95% CI, 37%–90%; p = 0.301) (Fig. 2). At last followup, six of 27 patients (22%) had died in the amputation group and nine of 52 (17%) had died in the microwave-induced hyperthermia group.

### Local Recurrence

With the numbers available, we found no difference in local recurrence (six versus 0; p = 0.066) between the amputation and microwave-induced hyperthermia groups. Six of the 52 patients who had microwave-induced hyperthermia (11.5%) (Table 1) had a local recurrence, whereas no patients in the amputation group had a local recurrence. The time to local recurrence was 4 to 18 months after surgery (median, 8.74 months). Two of the six patients were treated with microwave-induced hyperthermia again and four underwent amputations. No patient has had a second local recurrence.

### MSTS Functional Score

However, mean ± SD MSTS functional scores were higher in patients who had microwave-induced hyperthermia compared with those who had amputations (85% ± 6% versus 66% ± 5%; 95% CI of the difference, 16.01–23.10; p = 0.008) (Table 3). At latest followup, we observed no evidence of ankle instability, deformity, or degenerative changes of the ankle in any of the patients who had microwave-induced hyperthermia.

### Complications

With the numbers available, we found no difference in the proportion of patients experiencing postsurgical complications between the two groups (six of 52 [12%] versus three of 27 [11%]; odds ratio, 1.043; 95% CI, 0.240–4.544; p = 0.954). Complication severity, as graded according to Dindo et al. [3], likewise was not different with the numbers available (p = 0.9983). Six of the 52 patients who had microwave-induced hyperthermia (Table 3) experienced complications. Two patients experienced delayed union and eventually achieved union (Grade IIIb). One patient experienced fracture and was treated with arthrodesis (Grade IIIb). Two patients had superficial infections (Grade I), which resolved with local dressing changes. One patient had a deep infection (Grade IIIb), which was resolved by irrigation, débridement, and administration of systemic antibiotics.

Three of the 27 patients who had amputations (Table 1) experienced complications. Two patients experienced wound dehiscence and were treated with wound débridement (Grade IIIb). One patient had a superficial infection that resolved with local dressing changes (Grade I).

## Discussion

Below-knee amputation has been regarded as the standard surgical treatment for distal tibia osteosarcoma because of the difficulties in reconstruction when massive bone is lost so close to the ankle [16]. Historically, it has been very difficult to achieve satisfactory oncologic results and function with limb salvage in this anatomic location because of its particular challenges [12, 16, 18]. It has been reported that transtibial amputation provides a low risk of local recurrence and satisfactory function [2]. However, many patients refuse amputation for psychological or social reasons. Microwave-induced hyperthermia has been used with some success for two decades [7, 8]. We believe that the biggest advantage of microwave-induced hyperthermia is that it may relieve the patients of the need to have an amputation. However, to our knowledge, no comparative study has evaluated its efficacy for patients with distal tibia osteosarcoma. We therefore asked whether it would provide (1) improved survival, (2) decreased local recurrence, (3) improved MSTS scores, or (4) fewer complications than amputation in patients with a distal tibial osteosarcoma.

There were some limitations in this study. First, the sample size was relatively small despite this being one of the largest studies reported. This limited our ability to analyze for other factors that might have influenced the oncologic outcomes. Second, this study was a retrospective analysis and the two groups were not randomly selected. That being so, selection bias might have been an issue here. Patients perceived to have a worse prognosis may have been selected to have amputation. However, we tried to apply consistent indications for microwave-induced hyperthermia. In addition, the patients in whom limb salvage was not considered possible (such as those in whom neither the posterior tibial artery nor the dorsalis pedis artery was salvageable) were not included in any group. In general, patients were considered eligible for microwave-induced hyperthermia if they had an at least 20 mm available distance from the tumor edge to the articular surface, good clinical and imaging response to neoadjuvant chemotherapy, and no pathologic fracture. Patients not meeting these indications were treated with amputation. However, some patients meeting the indications for microwave-induced hyperthermia were treated instead with amputation or conventional limb-salvage approaches because of the patient’s subjective wishes (such as cost, function demand, social recognition). Two patients were unable to afford microwave-induced hyperthermia because of its high price and two other patients had anxiety owing to the possibility of tumor recurrence. Finally, the followup is relatively short. These patients need to be followed for longer periods to ensure that the tumors do not recur and that other complications related to treatment do not become evident. We intend to continue to follow these patients.

With the numbers available, we found no difference in oncologic survival between patients treated with microwave-induced hyperthermia and those who had transtibial amputation for distal tibia osteosarcoma. Other series [12, 16, 18] have had similar results between limb salvage and amputation for osteosarcoma of the distal tibia. However, the sample sizes in those studies are relatively smaller and comparisons were performed mostly between different types of reconstructions after limb salvage. Amputation is the secondary treatment when there is recurrence or a complication, in most cases.

Likewise, with the numbers available, the treatments were no different in terms of local recurrence, although there were some local recurrences in the microwave-induced hyperthermia group, and we believe that longer followup will be important in these patients. The incidence is relatively higher in other studies of limb salvage [5, 12, 16, 26], because it is difficult to obtain a safe margin resection when good function is desired at the same time because of the proximity of nerves, vessels, and tendons. When microwave-induced hyperthermia was given, the first step was to dissect the tumor-bearing bone from surrounding normal tissues with a safe margin. The distance between the ankle cartilage and the tumor as assessed by MRI was 20 mm or more to obtain a bone width margin of 10 mm and a remaining residual epiphysis of 10 mm. The margins of proximal bone resections were wide (a cuff of 2 cm to 3 cm of normal tissue remaining on all sides of the tumor). In addition, surrounding tissues were fully protected and multiple antennas were inserted in different locations from different angles to ensure the therapeutic range. This could account for some of the observed recurrence benefit of microwave-induced hyperthermia in our series. To the best of our knowledge, there were no local recurrences reported when amputation was performed, which is the same as in our study [5, 15, 18, 28, 29].

Our technique for microwave-induced hyperthermia resulted in improved function compared with transtibial amputation. Function is very important in all operations. However, the unfortunate reality is that better function seems to carry some risk of recurrence [2, 11, 14]. The reason for this is that for better function less tissue needs to be removed which could result in a high risk of recurrence. We also found that the mean MSTS functional scores for the patients who had microwave-induced hyperthermia were better than scores reported in other limb salvage studies [13, 23, 24]. There could be several reasons for this, although all are somewhat speculative. Osteotomy was not used, so the ankle remained intact; this could account for some of the observed functional benefit in this series. Second, we used a mixture of bone chips, cement, and prophylactic internal fixation for reconstruction. This may have facilitated revascularization, which has been confirmed by animal and clinical experiments [9, 15, 30], and perhaps helped to reduce the likelihood of nonunion, aseptic loosening, and allograft fracture. The maintained intraarticular structures can provide a good osseous bed for reattachment of resected soft tissues, such as muscles and ligaments.

Finally, we did not see an important difference between the treatment groups in terms of major complications. In fact, complications have a relatively high incidence in the distal tibia compared with other locations because of its unique anatomy [15, 19]. Reported complication rates range from 17% to 92% for patients having limb salvage treatment [16, 18, 27]. Topping that ranking were infection, allograft fractures, and nonunion, which is similar to our observed results.

Microwave-induced hyperthermia is an alternative treatment for distal tibia osteosarcoma, which in this series showed that it provided improved function compared with transtibial amputation, without any apparent increase in death, local recurrence, or complications. However, these findings should be replicated in larger studies with longer mean followups, and in studies that compare microwave-induced hyperthermia with conventional limb-sparing approaches.
